# Supplementary material for: Robust Formation of Ultrasmall Room-Temperature Neél Skyrmions in Amorphous Ferrimagnets from Atomistic Simulations
Source: Sci Rep. 2019 Jul 10;9:9964. doi: 10.1038/s41598-019-46458-4 (PMC6620327; doi:10.1038/s41598-019-46458-4)
Supplement: Supplementary file 1 — Supplementary Material for Robust Formation of Ultrasmall Room-Temperature Neél Skyrmions in Amorphous Ferrimagnets from Atomistic Simulations [file 41598_2019_46458_MOESM1_ESM.docx]

**Robust Formation of Ultrasmall Room-Temperature Neél** **Skyrmions in Amorphous Ferrimagnets from Atomistic Simulations**

**Chung Ting Ma^1^, Yunkun Xie^2^, Howard Sheng^3^, Avik W. Ghosh^1,2^, and S. Joseph Poon^1*^**

^1^Department of Physics, University of Virginia, Charlottesville, Virginia 22904 USA

^2^Department of Electrical and Computer Engineering, University of Virginia, Charlottesville, Virginia 22904 USA

^3^Department of Physics and Astronomy, George Mason University, Fairfax, Virginia 22030 USA

^*^ [sjp9x@virginia.edu](mailto:sjp9x@virginia.edu)

**Supplementary Material for** **Robust Formation of Ultrasmall Room-Temperature Neél** **Skyrmions in Amorphous Ferrimagnets from Atomistic Simulations**

*
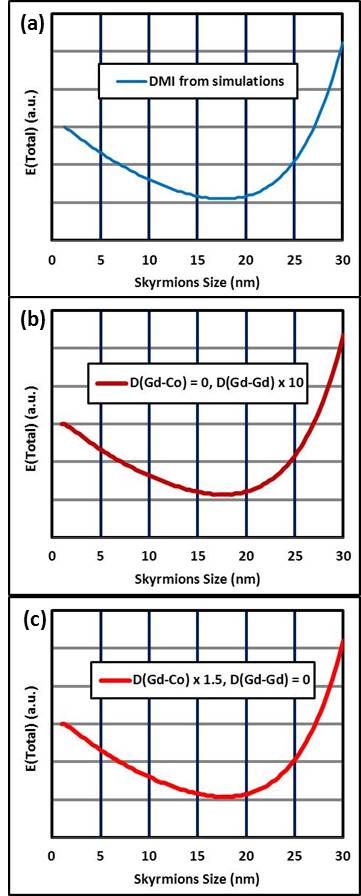
*

**Supplementary Figure 1. Energy landscape of 5 nm GdCo, with K = 0.3 x 10^5^ J/m^3^ and D = 0.4 mJ/m^2^. (a)** Using parameters from the simulations, energy minimum is at 18 nm skyrmion. **(b)** While average DMI is kept constant, D_Co-Gd_ is set to zero and D_Gd-Gd_ is increased by a factor of 10. Energy minimum is still at 18 nm skyrmion. **(c)** While average DMI is kept constant, D_Gd-Gd_ is set to zero and D_Gd-Co_ is increased by a factor of 1.5. Energy minimum remains at 18 nm skyrmion.

**Notes on Supplementary Figure 1.**

To study the effect of varying different DMI terms on the configurations, we employed the simulations results of 5 nm GdCo, with K = 0.3 x 10^5^ J/m^3^ and D = 0.4 mJ/m^2^, and incorporated the 2-pi model by Büttner, *el al*.^18^ to investigate the energy landscape. **Supplementary Figure 1 (a)** shows the energy landscape of the results shown in **Figure 5 (a).** The energy minimum of 18 nm skyrmions agrees with our results. **Supplementary Figure 1 (b) and (c)** show the energy landscape of two extreme cases, while total DMI is kept constant. In **(b)**, D_Co-Gd_ is set to zero and D_Gd-Gd_ is increased by a factor of 10 to compensate. In **(c)**, D_Gd-Gd_ is set to zero and D_Gd-Co_ is increased by a factor of 1.5 to maintain the total DMI. For both cases, energy minimum remains at 18 nm skyrmions. These show that no matter how the DMI terms are changed, as long as the total DMI remains the same, same skyrmion size remains the energy minimum.
